# Summer Buzz

**DOI:** 10.3201/eid1907.AC1907

**Published:** 2013-07

**Authors:** Polyxeni Potter

**Affiliations:** Centers for Disease Control and Prevention, Atlanta, Georgia, USA

**Keywords:** art science connection, emerging infectious diseases, art and medicine, Charles E. Burchfield, Summer Buzz, The Insect Chorus, vector-borne infections, mosquitoes, about the cover

**Figure:**
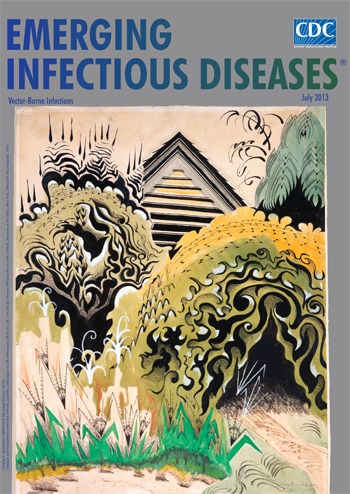
**Charles E. Burchfield (1893–1967) *The Insect Chorus* (1917) Opaque and transparent watercolor with ink, graphite, and crayon on off-white paper (50.8 cm × 38.1 cm)** Munson-Williams-Proctor Arts Institute, Museum of Art, Utica, New York, Edward W. Root Bequest, 1957

“It is late Sunday afternoon in August. A child stands alone in the garden listening to the metallic sounds of insects,” reads Charles Burchfield’s own description of *The Insect Chorus*. “They are all his world, so, to his mind, all things become saturated with their presence―crickets lurk in the depths of the grass, the shadows of the trees conceal fantastic creatures, and the boy looks with fear at the black interior of the arbor, not knowing what terrible thing might be there.”

*The Insect Chorus*, one of several works on the same theme, was painted during what Burchfield called his “golden year,” when he was fresh out of art school and back home in Salem, Ohio, in 1917. The work is a good example of his take on *en plein air* painting. An avid admirer of the outdoors, he immersed himself in it. He liked everything about it, even the annoying parts that most nature lovers merely tolerate for the sake of beauty. His artistic approach was a unique blend of naturalism, emotion, and imagination. “Surrounded by the familiar scenes of my boyhood, there gradually evolved the idea of recreating impressions of that period, the appearance of houses, the feelings of woods and fields, memories of seasonal impressions….”

Nature and its creatures remained of great interest to Burchfield all his life. He felt their sounds and captured them with extraordinary draftsmanship in his trademark watercolors. On the back of *The Song of the Katydids on an August Morning* (1917), he wrote, “A stagnant August morning during the drought season, as the pitiless sun mounts into the mid-morning sky, and the insect chorus commences, the katydids and locusts predominating. Their monotonous, mechanical, brassy rhythms soon pervade the whole air, combining with the heat waves of the sun, and saturating trees and houses, and sky.” Of *The August North (A memory of childhood)*, he wrote, “As the darkness settles down, the pulsating chorus of night insects commences swelling louder and louder until it resembles the heartbeat of the interior of a black closet.”

“Well, at one time I did think I was going to be a writer―when I was in high school and even the early years in art school I thought I was going to be a nature writer in somewhat the sense, you know, that Teale writes and Harold Borland, and so forth. And if I did anything with art work, I would make my own illustrations. But I soon realized that my outlook was visual rather than otherwise. But I mean it still… I did these short things.” He wrote impressions in his copious journals and descriptions on the back of his watercolors: “Crabbed old age sits in front of the black doorway without hope for the future, brooding. Spiders lurk in dark corners. The dying plants reflect her mood. The romantic outer moon rises just the same.” He even illustrated his own words, as in his notebook “Conventions for Abstract Thoughts,” his personal iconography, “A graphic shorthand of youth,” he called it, which came to be viewed by some as a visual vocabulary of American modernism.

Aside from colors and words, other forces also marked Burchfield’s output. “I get more out of music probably than I do out of other artists’ paintings, although I have many great admirations there,” he acknowledged. “It seems pertinent to me to insert here some thoughts on how interwoven music is with my painting. To many works, and even for whole periods of time, a definite piece of music or composition seemed to belong, though there might be no connection whatsoever between the music and what I was doing.”

Burchfield’s sensitivity to all that surrounded him nurtured his imagination and culminated in hundreds of works filled with a vibrant vision of the world, a vision in which humans and their creations were secondary and paled against the overpowering presence of nature. His style engaged whatever tools would bring forward this vision. “There are ideas that come to me that can be interpreted only in terms of patterns, and I derive much pleasure in working them out.” He defined his occupation in clear terms, “I like to think of myself—as an artist—as being in a nondescript swamp, up to my knees in mire, painting the vital beauty I see there, in my own way, not caring a damn about tradition, or anyone’s opinion.”

In *The Insect Chorus*, Burchfield paints summer in the outdoors, seen through the eyes of a child. The psychedelic spectacle transcends visual barriers to express the sweltering heat and humidity, along with the sounds of summer, its mystery and music. The alarming closeness of the clapboard household to the vegetation, which seems possessed by unknown forces, adds to the drama. Drooping ferns frame the canopy and its mysterious depths underneath the cool-color arabesques. Elaborate patterns―fluid lines, broad curves, cones―along with symbols to indicate sound and movement, transform a commonplace scene to a steaming hallucinogenic vision filled with emotion. V shapes in the grass mimic the jagged flight of crickets, and zigzag paths simulate their metallic sound, which Burchfield named “high shrill pin-point cricket chorus.”

By the early 1900s, mosquitoes were shown to transmit yellow fever and malaria from person to person, and human malaria was endemic in the United States. When Burchfield’s imaginary boy “looks with fear at the black interior of the arbor, not knowing what terrible thing might be there,” he is probably not worried about crickets alone but about all manner of vermin that you cannot see yet know are there. In the heat of a “Sunday afternoon in August,” mosquitoes awaiting a blood meal would be lurking in the vegetation. Along with a silent chorus of ticks, fleas, and rodents, they are part and parcel of the season and the locale.

Apart from nuisance and noise, the namesake insects of this painting rightly provoke fear. Many human pathogens infecting a large proportion of the world’s population and contributing to emerging disease are zoonotic and vector-borne. And many of these viruses, bacteria, protozoa, and rickettsia can be transmitted by blood-feeding arthropods. Depending on the geographic location, Burchfield’s vibrating canopy could play host to yellow fever, Rift Valley fever, Lyme disease, tularemia, malaria, typhus, Rocky Mountain spotted fever, to name only a few vector-borne infections that can begin with a summer buzz.

Vectors, such as mosquitoes, bridge barriers that would prevent transmission by direct contact between humans and especially between animals and humans. These barriers are not only spatial but also behavioral and ecologic. This ability of vectors includes moving a pathogen from one region to another. Because of the complex epidemiology and adaptiveness of pathogens and arthropods, vector-borne diseases are difficult to control, much less to eradicate. Vaccines are only available for a few diseases, and even when they are available, as for yellow fever, prevention can still be difficult to achieve.

Even previously successful strategies, such as pesticide impregnated bed-nets, can be compromised by human behavior and vector biology. With malaria, problems must be solved largely on the basis of local data because rarely do variables in one area behave in the same way as in another area, however closely the two regions may seem to resemble each other in topography and climate.

“How slowly the ‘secrets’ of my art come to me,” Burchfield lamented near the end of his life. He might as well have been expressing the epidemiologists’ frustration when they realize that changes in climate, land use, and transport, a concert eons in the making, affect rates of pathogen emergence in ways we still do not rightly understand. The artist’s respect for the all-encompassing power of nature applies across the board.
